# Gender, variation in opioid receptor genes and sensitivity to experimental pain

**DOI:** 10.1186/1744-8069-9-20

**Published:** 2013-04-09

**Authors:** Hiroe Sato, Joanne Droney, Joy Ross, Anne Estrup Olesen, Camilla Staahl, Trine Andresen, Ruth Branford, Julia Riley, Lars Arendt-Nielsen, Asbjørn Mohr Drewes

**Affiliations:** 1 Clinical Genomics group, Imperial College London, London, UK; 2 Department of Palliative Medicine, Royal Marsden Hospital, London, UK; 3 Mech-Sense, Department of Gastroenterology & Hepatology, Aalborg University Hospital, Aalborg, Denmark; 4 Center for Sensory-Motor Interactions (SMI), Aalborg University, Aalborg, Denmark

**Keywords:** OPRM, OPRK, OPRD, Gender, Pain tolerance thresholds, Opioid receptor genes

## Abstract

**Background:**

Pain tolerance is subject to considerable inter-individual variation, which may be influenced by a number of genetic and non-genetic factors. The mu, delta and kappa opioid receptors play a role in pain perception and are thought to mediate different pain modalities. The aim of this study was to explore associations between pain thresholds and gender and genetic variants in the three opioid receptor genes (*OPRM, OPRD* and *OPRK*). Experimental multi-modal pain data from previously published studies carried out in healthy Caucasian volunteers were used in order to limit the number of confounders to the study outcome. Data on thermal skin pain (n=36), muscle pressure pain (n=31) and mechanical visceral pain (n=50)) tolerance thresholds were included.

**Results:**

Nineteen genetic polymorphisms were included in linear regression modeling. Males were found to tolerate higher thermal and muscle pressure pain than females (p=0.003 and 0.02). Thirty four percent of variability in thermal skin pain was accounted for by a model consisting of *OPRK rs6473799* and gender. This finding was just outside significance when correction for multiple testing was applied. Variability in muscle pressure pain tolerance was associated with *OPRK rs7016778* and *rs7824175*. These SNPs accounted for 43% of variability in muscle pressure pain sensitivity and these findings remained significant after adjustment for multiple testing. No association was found with mechanical visceral pain.

**Conclusion:**

This is a preliminary and hypothesis generating study due to the relatively small study size. However, significant association between the opioid receptor genes and experimental pain sensitivity supports the influence of genetic variability in pain perception. These findings may be used to generate hypotheses for testing in larger clinical trials of patients with painful conditions.

## Introduction

Sensitivity to pain is subject to significant inter-individual variation. There has been growing interest in identifying the possible factors which might contribute to this variation. A significant confounder in the study of nociception in clinical practice is the existence of a number of different pain modalities including thermal, muscle pressure and visceral pain. Many clinically relevant pains such as cancer pain are likely to represent a mixture of these modalities. It is difficult, if not impossible to differentiate pain data from studies involving patients with painful disease in terms of pain perception modalities. Furthermore, nociception in patients with disease is influenced by a number of other factors including psychological state, concomitant medications, co-morbidities and disease treatment. Therefore studies which are carried out under experimental conditions in otherwise healthy volunteers may provide a “cleaner” platform from which to unpick inter-individual variation in pain perception.

Animal, familial and twin studies have provided convincing evidence of association between nociception and genetic variability. Painful conditions which appear to have some element of heritability include back pain, dysmenorrhoea, sciatica, musculoskeletal pain, spinal pain and irritable bowel syndrome [1-3]. Data from twin studies suggest up to 60% heritability of response to experimental painful stimuli [4,5]. There are also a number of heritable pain conditions in chronic non-cancer pain patients in which the underlying genetic mechanism has been identified [6]. One example includes the SCN9A gene, which codes for a peripheral sodium channel Na_V_1.7. Variation in this gene is associated with either a complete inability to sense pain or extreme pain disorders such as erythermalgia and paroxysmal extreme pain disorder [7,8]. Such disorders however are extremely rare. Although a number of “pain genes” have been proposed and analysed [9], to date the results of genetic association studies in clinically relevant painful conditions have been inconsistent, with little applicability to clinical practice. In most studies only one or a few polymorphisms in each possible pain gene have been analysed.

The mu, delta and kappa opioid receptors are all involved in nociception and are thus obvious candidates for a genetic association study. Recent data have suggested that these opioid receptors may be distinct in both physical location and also mode of pain transmission [10]. Only one candidate gene association study in experimental pain has examined genetic variation across all three opioid receptor genes [11]. That study was limited to women and explored only association with pressure pain. Furthermore only one polymorphism from OPRD and OPRK were included. The aim of this study was to investigate the association between multiple genetic variations (polymorphisms) across the mu, delta and kappa opioid receptor genes (*OPRM*, *OPRD* and *OPRK* respectively) and variability in sensitivity to multi-modal, multi-tissue experimental pain stimulations in healthy volunteers. Gender and age were also included in the statistical modeling as these factors are known to influence pain sensitivity [12,13].

## Results

### Study subjects

50 healthy Caucasian, opioid-naïve volunteers were included in this analysis; 22 female, 28 male, median age 26 years (range 19–48 years). Pain tolerance threshold median for thermal heat pain was 46.6°C (range 40.8-52.1), muscle pressure pain 39.4 kPa (range 25.5-76.4) and mechanical visceral pain 18.4 ml (5.2-67.8).

The intra-study reliability testing between baseline pain sensitivity scores demonstrated Cronbach’s α scores >0.7 for all pain modalities, confirming reliability. The average of the three baseline pain sensitivity scores were used as the outcome variables in stepwise linear regression analyses to explore associations with genetic factors and gender.

All SNPs (single nucleotide polymorphisms) were in Hardy-Weinberg equilibrium. The allele carriage frequencies in the study population are detailed in Table 1.

**Table 1:** Allele carriage frequencies in study population (n=50)

**Gene / SNP name**		**Location in gene**	**Amino acid change**	**Allele**	**Allele carriage frequencies**	
					**N**	**%**
*Opioid receptor mu (OPRM)*	*rs6912029*	Exon 1	*Gln-His*	*G*	50	100
				*T*	1	2
	*rs1799971*	Exon 1	*Asn-Asp*	*A*	49	98
				*G*	8	16
	*rs563649*	Intron 1		*C*	50	100
				*T*	9	18
	*rs9479757*	Intron 2		*G*	12	24
				*A*	49	98
	*rs533586*	Intron 2		*C*	31	62
				*T*	43	86
*Opioid receptor kappa (OPRK)*	*rs10504151*	Intron 2	-	*T*	50	100
				*C*	9	18
	*rs7836120*	Intron 2	-	*A*	49	98
				*G*	20	40
	*rs6473799*	Intron 2	-	*T*	45	90
				*C*	24	48
	*rs1365098*	Intron 2	-	*G*	43	86
				*T*	30	60
	*rs7016778*	Intron 2	-	*T*	50	100
				*A*	15	30
	*rs7824175*	Intron 3	-	*G*	47	94
				*C*	12	24
	*rs16918875*	Exon 4	*Val-Val*	*C*	50	100
				*T*	4	8
	*rs963549*	3UTR	-	*G*	47	94
				*A*	18	36
*Opioid receptor delta (OPRD)*	*rs1042114*	Exon 1	*Cys-Phe*	*G*	11	22
				*T*	50	100
	*rs533123*	Intron 1	-	*G*	15	30
				*A*	48	96
	*rs419335*	Intron 1	-	*A*	42	84
				*G*	23	46
	*rs2236857*	Intron 1	-	*T*	46	92
				*C*	15	30
	*rs2234917*	Exon 3	*Gly-Gly*	*C*	34	68
				*T*	40	80

### Pain and gender

**Figure 1:**
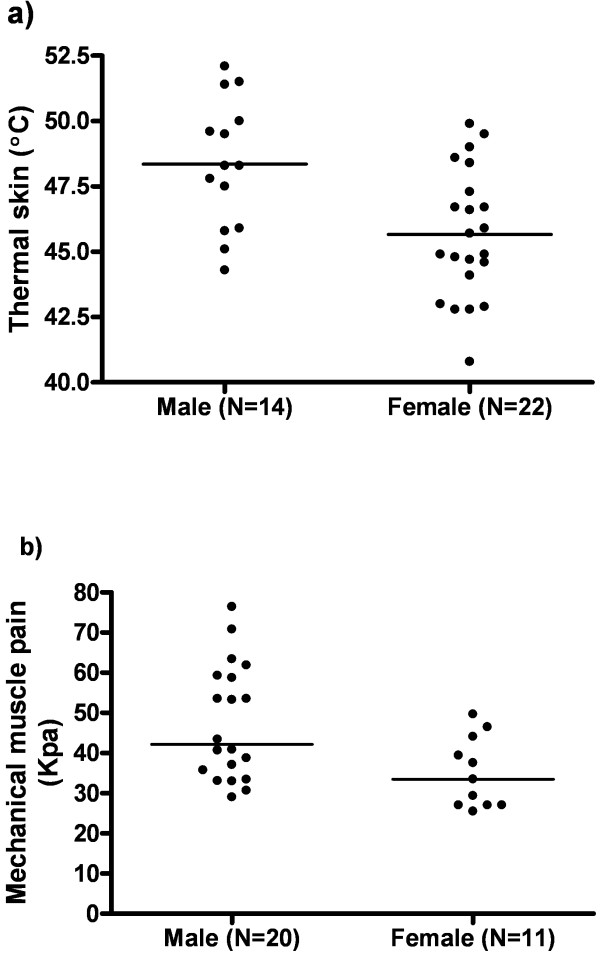
**Pain and gender. a**) Males (n=14, 48.4°C) have higher thermal skin pain thresholds than females (n=22, 46.7°C) (p=0.003). Line demonstrates mean values. **b**) Males (n=20, 47.4 kPa) have higher muscle pressure pain thresholds than females (n=11. 35.1 kPa) (p=0.02). Line demonstrates median values.

There was a significant association between gender and both thermal skin pain and muscle pressure pain. Males had higher pain thresholds in both thermal skin pain (male; n=14, average 48.4 +/− 2.5°C vs. female; n=22, average 46.7 +/− 2.5°C, p=0.003), (Figure 1a) and muscle pressure pain (male; n=20, median 47.4 kPa (range 29.0-76.4) vs. female; n= 11, median 33.5 kPa (range 25.5-49.7), p=0.02), (Figure 1b). Gender was not associated with variability in mechanical visceral pain.

### Pain and age

There was a significant association between muscle pressure pain and age (p=0.01). There was no association between age and thermal skin pain and mechanical visceral pain.

### Pain and the opioid receptors polymorphisms

#### Thermal skin pain

**Figure 2:**
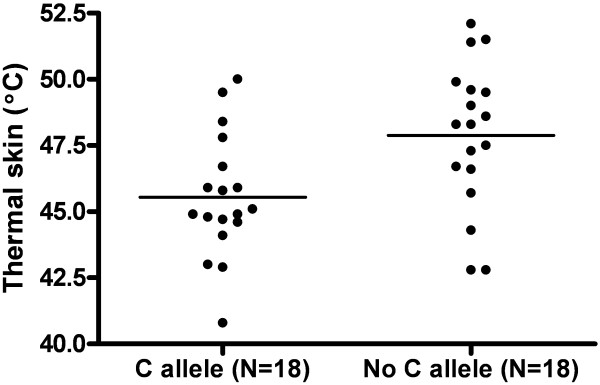
**Association between *OPRK rs643799 *and variability in thermal skin pain thresholds (univariate analysis).** Carriers of *OPRK rs643799C* allele (genotype *CC / CT*, n=18, 47.9+/−2.7°C) have lower thermal pain thresholds than non-carriers (genotype *TT*, n=18, 45.5+/−2.3°C), p=0.009. Line demonstrates mean values.

Six predictor variables (gender, 3 *OPRK* SNPs and *2 OPRM* SNPs) were significant at the level of 10% (p<0.1) on univariate analysis for thermal skin pain and these were entered into the multivariate models (Table 2 and Figure 2). Thirty-four percent of variability in sensitivity to thermal heat pain was associated with *OPRK rs6473799* and gender on multivariate regression (Table 3).

**Table 2:** Factors entered into the multivariate models (p<0.1 on univariate regression analysis) for thermal skin pain and muscle pressure pain

		**p-values**
**Thermal skin pain**	Gender	0.003
(N=36)	*OPRK rs6473799C*	0.009
	*OPRM rs589046T*	0.035
	*OPRK rs963549G*	0.041
	*OPRK rs1365098G*	0.07
	*OPRM rs9479757G*	0.086
**Muscle pressure pain**	*OPRK rs7016778 A*	0.003
(N=31)	Age	0.01
	Gender	0.012
	*OPRK rs7824175C*	0.018
	*OPRK rs7836120G*	0.096

**Table 3:** Multivariate regression models of factors predictive of inter-individual in pain sensitivity

**Thermal skin pain (N=36)**					
Model	R square		B (CI)	Beta	p-value
1	0.232	gender	2.696 (0.987-4.405)	0.482	0.003
2	0.339	gender	2.267 (0.613-3.921)	0.405	0.009
		*OPRK rs6473799 (CC/CT vs TT)*	−1.84 (−3.45 ~ −0.22)	−2.315	0.027
**Muscle pressure pain (N=31)**					
Model	R square		B (CI)	Beta	p-value
1	0.264	*OPRK rs7016778 (AA/AT* vs *TT)*	0.149(0.055 ~ 0.244)	0.514	0.003
2	0.427	*OPRK rs7016778 (AA/AT* vs *TT)*	0.145 (0.06 ~ 0.23)	0.5	0.002
		*OPRK rs7824175 (CC/CG* vs *GG)*	−0.159(−0.275 ~ −0.044)	−0.405	0.009

B represents the regression coefficient. This is the change in the dependent variable associated with a unit change in the predictor variable.

Beta represents the standardised regression coefficient.

The proportion of variability in the pain tolerance threshold that may be explained by its association with the factors in the regression model is calculated using R^2^.

When the Bonferroni adjustment for multiple testing was applied, p<0.008 for thermal skin pain and p<0.01 for muscle pressure pain were considered as significant.

When the Bonferroni adjustment for multiple testing was applied (p<0.008 was significant for thermal skin pain), these associations were just outside significance.

#### Muscle pressure pain

**Figure 3:**
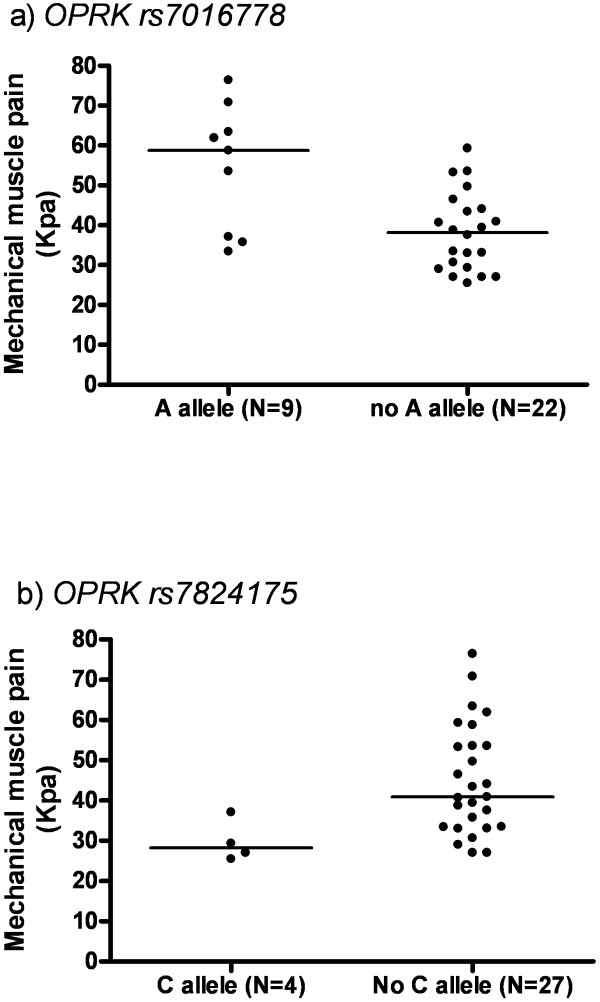
**Association between *OPRK *SNPs and variability in muscle pressure pain thresholds (univariate analysis). a**) Carriage of the *OPRK rs7016778 A* allele (genotype *AA / AT,* n=9, median 58.8 kPa (range 33.4-76.4)) had higher muscle pressure pain thresholds than non carriers (genotype *TT,* n=22, median 38.2 kPa (range 25.5-59.3*)*). p=0.008. **b**) Carriers of the *OPRK rs7824175 C* allele (genotype *CC / CG, n=4,* median 28.2 kPa (range 25.2-37.1)) had lower pain thresholds than non carriers (genotype *GG,* n=27, median 40.9 kPa (range 27.0-76.4)), p=0.01. Lines demonstrate median values.

Five predictor variables (gender, age, and 3 *OPRK* SNPs) were significant at the level of 10% (p<0.1) on univariate analysis for muscle pressure pain and these were entered into the multivariate models (Table 2 and Figure 3). *OPRK rs7016778* and *rs7824175* were retained as independent predictors of muscle pain sensitivity in multivariate regression analysis. These findings remained significant after Bonferroni correction for multiple testing (adjusted p<0.01 was significant for muscle pressure pain). Together these SNPs accounted for 43% of variability in sensitivity to muscle pressure pain (Table 3). In our data these SNPs are not in strong linkage disequilibrium (LD) (r^2^=0.03, LOD=0.55), mirroring the LD parameters from the HapMap Project [14]. Age and gender were not retained as significant predictors of muscle pressure pain in regression analysis.

#### Mechanical visceral pain

This parameter was not significantly associated with gender or any polymorphisms in *OPRM, OPRD* or *OPRK*.

Haplotype analysis did not add to the associations described.

## Discussion

This is the first pain genetics study in which multiple polymorphisms across all three opioid receptor genes (*OPRM, OPRD, OPRK*) were considered together to build a predictive model of nociception. These data suggest that genetic factors and gender account for 34-43% of variability in thermal and mechanical pain sensitivity.

### Opioid receptor genes and pain

As opioid receptors are key players in the nociceptive pathway, the genes coding for these receptors were chosen as candidates in this study. Most previous genetic association studies in this area have focused on *OPRM*. A number of different polymorphisms across *OPRM* have been associated with variability in pain sensitivity but there have been a number of inconsistencies between studies [15] and the clinical significance of the findings is unclear In humans there is much less data regarding the influence of polymorphisms in *OPRD* and *OPRK* in pain sensitivity. In a study of 500 normal participants, a polymorphism in *OPRD rs2234918* was associated with a gender specific difference in thermal pain sensitivity [16]. However *OPRD* haplotype analysis in a similar study by the same researchers did not show any association [17]. Although one study in mice found an association between OPRK and morphine antinociception [18], this is the first study in humans to suggest such a relationship.

In our study we found associations between the opioid receptor genes and thermal and muscle pressure pain but not with mechanical visceral pain. It may be that different opioid receptors have different influences on discrete pain modalities. The mu-opioid receptor (MOR) is thought to be involved in perception of thermal and chemical pain [19,20]. The delta-opioid receptor (DOR) plays a role in mechanical, neuropathic and inflammatory pain [19,21]. Although the data are controversial, some authors suggest that the kappa opioid receptor (KOR) might be involved in mediating visceral pain [19,22]. In our data, no *OPRM* SNPs were retained as independent predictors of pain sensitivity to any of the pain modalities. Instead, polymorphisms in *OPRK* were associated with thermal and mechanical pain. OPRK was not found to be associated with visceral pain perception. The functional significance of the OPRK SNPs is unknown. It is unclear why *OPRK* rather than *OPRM* or *OPRD* appears to influence pain sensitivity. One possible mechanism may be through the process of receptor dimerisation. Opioid receptor subtypes MOR, DOR and KOR are thought to interact with each other to form complexes known as receptor dimers, which may have altered function [23,24]. Therefore genetic variability in OPRK, the gene coding for KOR, may indirectly influence the role of MOR or DOR in pain processing. This is an area of controversy however as more recent data have suggested that opioid receptors, especially MOR and DOR, may be distinct in both physical location and also mode of pain transmission [10].

This is the first study to test and find associations between experimental pain sensitivity and polymorphisms in *OPRK*. In this study *OPRK rs7824175* was associated with variability in mechanical muscle pain. We have previously identified an association between *OPRK rs7824175* and residual pain in a cohort of cancer patients titrated on oral morphine [25].

The *OPRK* SNPs which are associated with variation in pain sensitivity in this study are found in intronic regions of the gene. Introns are generally not involved in coding for protein synthesis. The exact role of these intronic SNPs in opioid response is not yet known. It is possible that they may influence clinical outcome through a process known as linkage disequilibrium (non-random association), whereby these polymorphisms may represent markers for true susceptibility polymorphisms [26] which do alter protein function. This may explain why SNPs which are not in linkage disequilibrium together remained significant on multivariate testing. Alternatively these intronic SNPs may be involved in the production of functionally variable opioid receptor subtypes (isoforms) through the mechanism of alternative pre-mRNA splicing. Practical challenges of examining human brain RNA means that most research into gene expression in this area has been carried out in vitro using cell lines expressing mu opioid receptors [27]. There are data however to suggest that genetic variation at the RNA level does occur. Only one *OPRM* gene has been identified yet at least 25 splice variants from the mouse *OPRM* gene have been identified and 11 from humans [28]. An intronic SNP, OPRM rs563649 has been associated with altered mRNA levels and translation efficacy in OPRM isoforms [29]. Alternative splicing for the delta and kappa opioid receptor have also been detected [30].

It is likely that pain perception is under the control of a number of different interacting genetic and environmental influences [31]. Despite minimising the number of non-genetic confounders by carrying out these analyses in healthy volunteers, the data presented in this paper demonstrate that there remain a number of other factors which are likely to significantly contribute to variability in pain sensitivity. Gender accounted for approximately 20% of the variance in thermal pain sensitivity. The finding that males were able to tolerate higher somatic but not visceral pain thresholds mirrors other studies [12,32,33]. Hormonal differences, differences in skin thickness between males and females or neurobiological factors may play a role [33,34].

### Limitations

As with any genetic association study in pain, there are a number of limitations. This study explored the association between opioid receptor genes and experimental pain sensitivity. It carried out in healthy Caucasian volunteers, with uncertain relevance to patients with painful conditions or subjects with different ethnicities. It is likely that there are many more “pain genes” which contribute to variability in pain sensitivity, each with a relatively small effect size. For example, serotonin is a key signaling molecule in the gastrointestinal tract and is involved in both pro-nociception and antinociception [35]. Polymorphisms in genes involved in the serotonergic system such as *SLC6A4*, which codes for the serotonin re-uptake transporter, may be influential in visceral pain perception. Similarly, it is likely that other non-genetic factors affect nociception. For example, there is documented association between pain (particularly visceral pain) and psychometric indices such as anxiety and neuroticism [36-38]. There are also studies which suggest that sleep, psychological distress and blood pressure can alter pain perception [39,40]. These parameters were not measured in this study. Different experimental pain modalities have been shown to be poorly correlated, suggesting that they represent different specific dimensions of pain perception [41]. Therefore it is likely that different genetic and non-genetic factors are associated with each pain modality, as suggested by our data. Larger studies are required to test all genes and all gene-gene and gene-environment interactions.

The power of genetic association studies and thus the sample size required to detect true associations is dependent on a number of factors including the number of polymorphisms being tested, the frequency of the susceptibility allele, the strength of the association and the complexity of the trait being examined. This study was specifically designed to minimise the impact of some of these factors. Accurate phenotype definition has yielded significant results in relatively small studies previously. One of the landmark studies of the role of UGT1A1 in irinotecan toxicity included only 63 patients, of whom six had the clinical outcome of interest (grade 4 neutropenia)[42]. One of the earliest genome wide association studies was carried out in 146 individuals (96 cases and 50 controls) in which 103,611 SNPs were analysed and a polymorphism in the gene coding for complement factor H was found to be significantly associated with advanced age-related macular degeneration [43]. This phenotype was later tightened a GWA conducted in 96 cases and 130 controls yielded very significant results (p = 4.1 × 10–12) which have been subsequently replicated [44]. Pain perception is a complex trait, genetic variability is likely to be associated with small or modest effect sizes [45]. In this study, unlike genetic association studies in patients undergoing painful procedures or patients with cancer pain, pain stimulation was standardised and reliable, objective accurate study outcomes were used and the study population comprised of otherwise healthy volunteers. Therefore the number of potential confounding factors and the complexity of the outcomes being measured were minimised. In this study SNPs were only included if they had a minor allele frequency of > 10%. In this study the genetic associations with muscle pressure pain remained significant even after conservative correction for multiple testing. However, a formal pre-study power calculation was not performed because the patient data were originally collected for different studies. Therefore the findings presented in this study must be considered preliminary and hypothesis generating. These findings need to be replicated in other studies and larger sample sizes are undoubtedly required to identify the smaller genetic effect sizes [46].

Finally, experimental pain stimuli cannot be compared directly to pain in the clinical settings where hyperalgesia and a variety of psychosocial variables come into play. However, the current models have been shown to be translatable to clinical pain [47,48] and the data presented here may be used to generate hypotheses for testing in clinical trials of patients with painful conditions.

## Conclusion

Although this is a small study, significant association between the opioid receptor genes and experimental pain sensitivity supports the influence of genetic variability in pain perception. Experimental pain studies in healthy volunteers provides a platform from which to explore inter-individual variation in sensitivity to pain, which is relatively free from confounders that limit studies in painful disease states. Replication in larger studies involving patients is necessary to further explore this association and to examine the clinical significance of such findings.

## Methods

Data were collected from three previously published experimental pain studies in healthy volunteers. The first study (Study A) was a comparative study of oxycodone and morphine in a multi-modal, tissue-differentiated experimental pain model [49]. The second (Study B) was a study of analgesic efficacy of peripheral κ-opioid receptor agonist CR665 compared to oxycodone in a multi-modal, multi-tissue experimental human pain model [50]. The third (Study C) was a study of different effects of morphine and oxycodone in experimentally evoked hyperalgesia [47]. All studies were approved by the local Ethical Committee (A: VN 2002/143, B: VN-20060021, C: N-0070025) and The Danish Medicines Agency (A: 2612–2168, B: 2612–3145, C: 2612–3463).

Each study was a three-arm study, with each subject receiving placebo, oxycodone and either morphine or CR665. Therefore each subject underwent baseline pain sensitivity testing on three occasions, each at least one week apart. Only these baseline pain data, which were assessed before administration of placebo and each of the two study drugs, were included in this study. This study includes data from pain sensitivity tests which were performed in at least two out of the three studies. Thermal skin pain, muscle pressure pain and mechanical visceral pain were analysed. For each study subject, details about age and gender were also recorded.

DNA was available for 19 out of 24 subjects from Study A, 15 out of 18 subjects from Study B and 21 out of 24 subjects from Study C. One subject from Study B was excluded due to non-Caucasian ethnicity, in order to minimise bias due to population stratification in the genetic association analysis. Four subjects were excluded from study C as they had already participated in Study B.

### Experimental pain sensitivity testing

#### Thermal skin pain

Thermal skin testing data were used from studies A and C (total N = 36). A computer-driven heat pain device (TSA-II NeuroSensory Analyzer, Medoc Ltd, Ramat Yishai, Israel) was used for the heat stimulation. A thermode with a surface of 25 × 50 mm was applied to the volar surface of the forearm 10 cm distal from the elbow and the subjects were asked to press a button when the pain tolerance threshold (PTT) was reached i.e. when they could no longer withstand the pain. The temperature was increased from 32°C to a maximum at 52°C at a rate of 1°C/s. When the subjects pressed the button, the thermode was cooled to 32°C and the experiment repeated. Three consecutive measurements were performed and the average was computed.

#### Muscle pressure pain

Muscle pressure pain data were used from studies B and C (total N = 31). The electronic cuff algometer (Aalborg University, Aalborg, Denmark) consisted of a pneumatic tourniquet cuff, a computer-controlled air compressor, and an electronic 10-cm visual analogue scale (VAS). The compressor (Condor MDR2; JUN-AIR International A/S, Nørresundby, Denmark) was connected to an electric-pneumatic converter (ITV2030; SMC Corp., Tokyo, Japan) and controlled by a computer through a data acquisition card (PCI 6024E; National Instruments, Austin, TX). The pain intensity was recorded continuously on the visual analogue scale (VAS) and sampled at 100-ms intervals. The pneumatic tourniquet cuff was wrapped tightly around the gastrocnemius muscle. The cuff was automatically inflated (compression rate 0.50 kPa/s) until the PTT was reached.

#### Mechanical visceral pain

Mechanical visceral pain data were used from studies A, B and C (total N = 50). A probe designed for multimodal stimulation of the oesophagus was used. Before the study all subjects were instructed how to use the 0–10 electronic VAS, for the visceral stimulations, where 0 = no perception and 10 = unbearable pain. In order to induce mechanicaloesophageal pain, the oesophageal bag was distended at a constant infusion rate until ‘moderate pain’ intensity ratings (defined in these studies as VAS score of 7) were reached. The volumes (ml) of distension at this point were used for further analysis.

### Single nucleotide polymorphism (SNP) genotyping

Genotyping for polymorphisms across *OPRM*, *OPRK* and *OPRD* was carried out using sequence specific primers and polymerase chain reaction. Five polymorphisms in *OPRM (rs6912029G/T, rs179997A/G, rs56364C/T, rs9479757G/A, rs533586C/T)*, eight in *OPRK (rs1050415T/C, rs7836120A/G, rs647379T/C, rs1365098G/T, rs701677T/A, rs7824175G/C, rs16918875C/T, rs963549G/A)* and five in *OPRD (rs1042114G/T, rs533123G/A, rs419335A/G, rs2236857T/C, rs2234918C/T)* were included in the study. The details of primers are as previously described [25]. Within each of the genes polymorphisms were selected to try to cover allelic diversity across each gene. Polymorphisms in regions most likely to have an impact on gene function were prioritised (promoter region, exons, intron-exon boundaries, 3-UTR), as were polymorphisms for which there exists published data.

Linkage disequilibrium between pairs of SNPs was tested and haplotypes were constructed using the software programmes PHASE [51] (http://www.stat.washington.edu/stephens/software.html) and Haploview (Haploview version 4.0, Broad Institute, Cambridge, USA) [52].

### Statistical analysis

All genotype frequencies were tested for Hardy-Weinberg equilibrium using Chi-square goodness-of-fit test (Haploview version 4.0, Broad Institute, Cambridge, USA). Each genetic variant tested was a bi-allelic single nucleotide polymorphism. The genetic model under which a genetic variant may be assumed to influence the pain sensitivity phenotype is unknown. It is not known whether the variant allele could be a risk-enhancing allele (increases the likelihood of a poor outcome) or a protective allele (increases the likelihood of a good outcome). For this reason allele carriage, frequency of subjects homozygous (AA) + heterozygous (AB) for allele A versus frequency of subjects homozygous (BB) for allele B, was used for the genetic association analysis (Table 1).

Reliability between the three baseline scores for each patient was tested using Cronbach’s α. Values >0.7 were considered to be indicative of reliability. The average of the three baseline pain sensitivity scores were used as the outcome variables in the genetic association study.

A number of statistical methods for examining gene-gene and gene-environment interactions have been proposed including regression, classification and regression trees, neural networking, combinatorial partitioning and multifactor dimensionality reduction [53]. In this study multivariate linear regression was used to investigate the joint effect of the predictor variables (clinical and genetic) on the experimental pain thresholds (dependent variables).

Univariate analyses were carried out to screen for an association between individual clinical (gender and age) and genetic predictor variables and the outcome variables (average pain sensitivity scores). Normally distributed continuous variables (thermal skin pain and mechanical visceral pain) were analyzed using t-tests and non-parametric data (muscle pressure pain) were analysed using Man-Whitney U test. Factors with p<0.1 on univariate analysis (Table 2) were included in the multivariate modelling. Variables with p>0.1 were excluded in order to reduce the number of predictor variables. Variable selection was carried out using a stepwise method. Only factors with p<0.05 were retained in the final model. Non-parametric outcome data (i.e. muscle pressure pain) were logarithmically transformed for regression analysis. A Bonferroni adjustment for multiple testing was applied, dependent on the number of factors included in the final modelling [54].

Statistical analysis and plots were performed using PASW 18 for Windows (SPSS, Chicago, IL) and GraphPad version 4.02 for Windows (GraphPad Software, San Diego California USA).

## Abbreviations

OPRK: Opioid receptor kappa; OPRD: Opioid receptor delta; SNP: Single nucleotide polymorphism; LD: Linkage disequilibrium; MOR: Mu-opioid receptor; DOR: Delta-opioid receptor; KOR: Kappa opioid receptor; VAS: Visual analogue scale

## Competing interests

The authors declare that they have no competing interests.

## Authors’ contributions

HS, JD, JRR and RB designed and carried out the genetic assays and analyses. HS and JD carried out the statistical analyses and drafted the manuscript. AMD and JR conceived of the study and participated in its coordination. AMD, AEO, CS, TA and LAN carried out the pain tolerance threshold testing and analysis. All authors were involved in reading, amending and approving the final manuscript.
